# Mechanism of Jiawei Zhengqi Powder in the Treatment of Ulcerative Colitis Based on Network Pharmacology and Molecular Docking

**DOI:** 10.1155/2023/8397111

**Published:** 2023-02-20

**Authors:** Chao Zhao, ChenYang Zhi, JianHua Zhou

**Affiliations:** ^1^College of Traditional Chinese Medicine, Changchun University of Chinese Medicine, Changchun, China; ^2^Anorectal Diagnosis and Treatment Center, Affiliated Hospital of the Changchun University of Chinese Medicine, Changchun, China

## Abstract

**Objective:**

Ulcerative colitis is an intestinal condition that severely affects the life quality of a patient. Jiawei Zhengqi powder (JWZQS) has some therapeutic benefits for ulcerative colitis. The current study investigated the therapeutic mechanism of JWZQS for ulcerative colitis using a network pharmacology analytical approach.

**Methods:**

In this study, network pharmacology was used to investigate the potential mechanism of JWZQS in treating ulcerative colitis. The common targets between the two were identified, and a network map was created with the Cytoscape software. The Kyoto Encyclopedia of Genes and Genomes (KEGG) and Gene Ontology (GO) enrichment analyses of JWZQS was performed using the Metascape database. Protein-protein interaction networks (PPI) was created to screen core targets and main components, and molecular docking was conducted between the main components and core targets. The expression levels of IL-1*β*, IL-6, and TNF-*α* were detected in animal experiments. Their effect on the NF-*κ*B signaling pathway and the protective mechanism of JWZQS on the colon by tight junction protein were investigated.

**Results:**

There were 2127 potential ulcerative colitis targets and 35 components identified, including 201 non-reproducible targets and 123 targets shared by drugs and diseases. Following the analysis, we discovered 13 significant active components and 10 core targets. The first 5 active ingredients and their corresponding targets were molecularly docked, and the results showed a high level of affinity. GO analysis showed that JWZQS participate in multiple biological processes to treat UC. KEGG analysis showed that JWZQS may be involved in regulating multiple pathways, and the NF-*κ*B signaling pathway was selected for analysis and verification. JWZQS has been shown in animal studies to effectively inhibit the NF-*κ*B pathway; reduce the expression of IL-1*β*, TNF-*α*, and IL-6 in colon tissue; and increase the expression of ZO-1, Occludin, and Claudin-1.

**Conclusion:**

The network pharmacological study provides preliminary evidence that JWZQS can treat UC through multiple components and targets. JWZQS has been shown in animal studies to effectively reduce the expression levels of IL-1*β*, TNF-*α*, and IL-6, inhibit the phosphorylation of the NF-*κ*B pathway, and alleviate colon injury. JWZQS can be used in clinical, but the precise mechanism of UC treatment requires further investigation.

## 1. Introduction

Ulcerative colitis (UC) is an inflammatory condition that primarily affects the mucosa and submucosa of the large intestine (rectum and colon). It is distinguished by large intestinal mucosal ulceration and persistent inflammation [1]. Mucous, diarrhea, hemafecia, pus, stomachache, and tenesmus were the most common clinical symptoms [2]. Medication, surgery, and antibody therapy are the three main types of current UC treatments. The most common existing therapies include aminosalicylates, corticosteroids, and immunosuppression, all of which have negative side effects and necessitate long-term medication [3]. Therefore, safer and more effective therapeutic approaches for UC are required.

In traditional Chinese medicine (TCM), there are several ways to treat UC, which is also known as diarrhea or dysentery. TCM provides a comprehensive understanding of a patient's physical condition based on their pulsation, tongue, signs and symptoms, and modern laboratory tests. Although therapeutic regimens vary depending on constitution, the general approach is to clear heat; expel dampness; strengthen the spleen, kidney, and qi; promote blood circulation; and dispel blood stagnation. JWZQS decoction contains Houpo, Chenpi, Banxia, Danggui, Chuanxiong, Huoxiang, Cangzhu, Xianhecao, and Shengma. The original prescription comes from a Ming Dynasty work by Xue Ji called the “Waike Jingyao.” It has Qi-widening, spleen-energizing, and dampening effects. The current study makes reference to Andrew L. Hopkins' theory of “network pharmacology” and Professor Li Shao's theory of “correlation between TCM and biomolecular network” [4, 5]. Network pharmacology, which combines pharmacology and systems biology, is an analytical system suitable for the study of complex components of traditional Chinese medicine.

**Figure 1:**
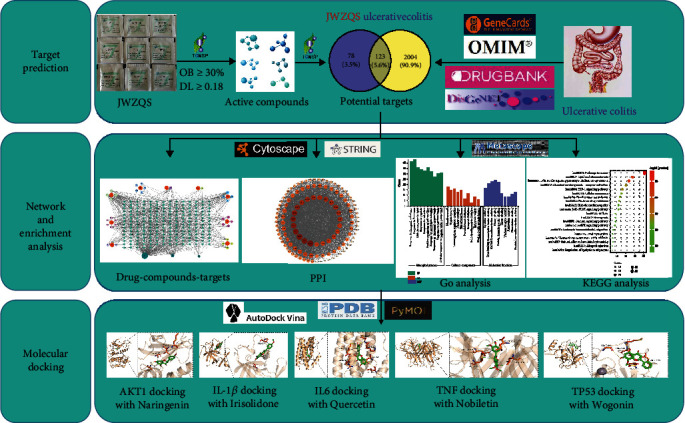
Flow chart representing anti-UC network pharmacology of JWZQS.

The current study used the network pharmacology approach to construct the “drug-components-targets” network. For the core target and pathway verification, the KEGG and GO enrichment analyses were performed. Molecular docking technique was used to study the affinity between active components and key targets in JWZQS. The flow chart for the study is shown in Figure 1.

We used dextran sulfate sodium salt (DSS) to cause colon injury in mice, developed a UC mouse model, and assessed the clinical symptoms of the mice. Mouse colons were collected, colonic lengths were measured, and the contents and expressions of TNF-*α*, IL-6, and IL-1*β* in tissues were measured. The phosphorylation of I*κ*B-*α* and P65 in the NF-*κ*B pathway was detected. Furthermore, the tissue was sliced to examine the damage to the colon. Immunohistochemistry (IHC) detected the expression of ZO-1, Occludin, and Claudin-1 in the mouse colon tissue.

## 2. Article Types

The article type was original research.

## 3. Methods

### 3.1. Collection of Components and Targets of JWZQS

We use the search terms “ Houpo, Chenpi, Banxia, Danggui, Chuanxiong, Huoxiang, Cangzhu, Xianhecao, and Shengma” in the TCM database of system pharmacology (TCMSP, http://tcmspw.com). Following the bioactive constituents criteria, active constituents with drug similarity (DL) not less than 0.18 and oral bioavailability (OB) not less than 30% were screened [6]. Based on TCMSP, we identified potential targets of active JWZQS constituents. Using the UniProt database, species were restricted to “Homo sapiens,” and annotation normalization was applied to the target genes [7].

### 3.2. Identification and Collection of Disease Gene Targets

TGenecards (https://www.genecards.org), OMIM (https://www.omim.org), DrugBank (https://go.drugbank.com), and DisGeNet (https://www.disgenet.org) databases were searched for UC, and human genes were chosen [8–11]. After collecting disease-associated genes from the four databases, duplicate values were removed, yielding 2127 disease-related gene targets. Drugs and diseases may have similar targets for disease management [12]. String database (https://string-db.org) was used to screen out the common targets.

### 3.3. Construction of Drug-Components-Targets Network

Cytoscape software (3.10.1 version) is used to associate drugs, active ingredients, targets, and diseases and to build an interaction network diagram between components and targets. Edges represent node relationships, while nodes represent active ingredients and targets. The main active components of the drugs were then examined.

### 3.4. Construction of PPI Network and Screening of Core Targets

To establish the interaction between the two proteins, the common target was imported into the Cytoscape database (https://cytoscape.org), and restriction conditions were set for the human species [13]. The database calculated the interrelationship, closeness, degree, feature vector, average local connectivity, and network for network analysis. Finally, the traditional “degree” value was employed to assess the importance of PPI network nodes.

### 3.5. Analysis of Enrichment and Associated Signaling Pathways

To study JWZQS's biological process and signaling pathway in managing UC, we used the Metascape website to analyze molecular function (MF), biological process (BP), and cell component (CC) data. Their sequencing is determined by the number of targets involved in biological processes and signaling pathways.

### 3.6. Molecular Docking

We downloaded the protein crystal architectures used for docking from the PDB database (https://www.rcsb.org) in addition to the 3D structures of the small molecules irisolidone, naringenin, nobiletin, quercetin, and wogonin from the PubChem (https://pubchem.ncbi.nlm.nih.gov) database [14].

AutoDock Vina 1.1.2 was used in the current study to perform molecular docking [15]. PyMol 2.5.4 was used to remove small molecules, salt ions, and water molecules from the receptor proteins before docking [16]. A docking box was created to contain the entire protein architecture. The entire processed receptor proteins and small molecules were converted into the PDBQT format via ADFR suite 1.0 to prepare AutoDock Vina 1.1.2 docking [17]. While connecting, keep the default settings for interconnection parameters. The binding confirmation was determined to be the output docking confirmation with the highest score. The final step involved a visual analysis based on PyMol 2.5.4 docking results.

### 3.7. Animal Experiment

Animal experiments were carried out following relevant legal principles and were approved by the Institutional Animal Management and Use Committee of Jilin University (Changchun, China) according to the procedures (License No.: SYXK2018–0001). Suzhou Xishan Biotechnology Co. LTD. provided male Kunming mice (6~8 weeks old). Thirty Kunming mice were randomly divided into five groups: the blank group (NT), the model group (MOD), the sulfasalazine group (SASP), the JWZQS low-dose group (JWZQS-L), the JWZQS medium-dose group (JWZQS-M), and the JWZQS high-dose group (JWZQS-H). Each group had five mice. For UC model establishment, except for the control group, the other mice were given 3% DSS solution for 7 days, according to the literature [18].The drugs studied in this experiment were obtained from the granule Pharmacy of the Affiliated Hospital of the Changchun University of Chinese Medicine. Houpo, Chenpi, Banxia, Danggui, Chuangxiong, Huoxiang, Cangzhu, Xianhecao, and Shengma were modulated in a ratio of 1.5 : 1.5 : 1.5 : 2 : 1 : 1.5 : 1 : 3 : 1 (batch numbers: 21097204, 21087554, 21076314, 21096634, 21106334, 21077654, 21097014, 21096254, and 21078184). According to the experimental drug dose recommended by FDA (Food and Drug Administration), the equivalent dose of mice (11.7 g/kg) was 9.0 times that of humans (93 g original drug/70 kg body weight = 1.3 g/kg). The equivalent dose was set as the JWZQS-M group (11.7 g/kg). The JWZQS-H group (23.4 g/kg) had twice the concentration as the JWZQS-M group, and the JWZQS-L group (5.85 g/kg) had 0.5 times the concentration as the JWZQS-M group. The SASP (Shanghai Xinyi Co. LTD.) group 100 mg/kg SASP suspension by gavage [19]. Gavage was used to administer the corresponding volume of distilled water to the NT group.

### 3.8. Clinical Scoring and Sample Collection

The disease activity index (DAI) scoring system (Table 1) was used to determine body weight (BW), fecal occult blood, fecal characteristics, and clinical symptoms. Finally, colons of mice were collected for relevant experimental analysis [20].

### 3.9. ELISA

TTNF-*α*, IL-1*β*, and IL-6 expression levels in mouse colon tissues were measured using the ELISA kit (ELISA MAX Deluxe Set Mouse IL-1*β* 432604, MAX Deluxe Set Mouse IL-6 431315, and ELISA MAX Deluxe Set Mouse TNF-*α*430915).

### 3.10. qRT-PCR Assay

Colon tissues from mice were collected and treated to extract mRNA, and the levels of IL-1*β*, TNF-*α*, and IL-6 were determined using qRT-PCR as previously described [21]. Colon tissues from mice were collected and treated to extract mRNA, and the levels of IL-1*β*, TNF-*α*, and IL-6 were determined using qRT-PCR as previously described [21].  Table 2 lists all of the substrates that were used. The arithmetic formula 2^−*ΔΔ*CT^ was used to obtain the relative quantitative results.

### 3.11. Western Blotting (WB) Assay

The total protein of colonic tissue was extracted in this study by grinding RIPA lysate. BCA (Thermo) reagent was used to measure colonic histamine levels in mice (Sevier Biotechnology, Wuhan, China). SDS-PAGE with 12% was used to isolate the protein (15 *μ*g), which was then transferred to a PVDF membrane (Millipore, Darmstadt, Germany). The PVDF membrane was then blocked for 2 h at room temperature with 5% skim milk. PVDF membrane and primary antibody NF-*κ*B p65 (Servicebio GB11997 1 : 300), NF-*κ*B p-p65 (Servicebio GB111421 1 : 300), I*κ*B-*α* (Abcam EPR20992 1 : 2000), p-I*κ*B-*α* (ser32 1 : 2000), and GAPDH (Servicebio GB15002 1 : 2000) were stored overnight at 4°C and washed with TBST lotion. The PVDF membrane was then washed in TBS-T washing solution and incubated for 2 h with goat anti-mouse/rabbit secondary antibody (Servicebio GB25301/GB23303 1 : 5000). The trend of protein bands was then detected using an enhanced chemiluminescence solution.

### 3.12. Hematoxylin and Eosin (H&E) Staining

Tissues from mouse colons were fixed in 4% formaldehyde and paraffin before being sliced into 5 *μ*m sections for H&E staining. After staining, the histology was examined under a light microscope. Finally, histological scores were computed following Table 3 [22].

### 3.13. Immunohistochemistry (IHC)

Immunohistochemical analysis of mouse colon tissue was performed to observe the contents of ZO-1, Occludin, and Claudin-1 proteins in mouse colon tissue to further understand the protective mechanism of JWZQS on mouse colon. The sections were incubated in a citrate antigen repair solution for 20 min at 95°C. These sections were incubated overnight with the primary antibodies ZO-1 (D6L1E 1 : 100), Occludin (ab216327 1 : 100), and Claudin-1 (ab15098 1 : 100) and then with the secondary antibodies for 50 min before being examined under a microscope.

### 3.14. Statistical Analysis

The data is presented as the mean ± SD. Prism 8.0 was used to compare two groups using unpaired Student's *t*-tests. Prism 8.0.20 was used to perform ANOVA (general linear model) comparisons of more than two groups. Nonparametric tests must be used to evaluate clinical and pathology scores.

## 4. Results

### 4.1. Collection of Active Components of JWZQS and Study of Overlapping Targets

The following nine Chinese medicines were searched in the TCMSP database: Houpo, Chenpi, Banxia, Danggui, Chuangxiong, Huoxiang, Cangzhu, Xianhecao, and Shengma. These compounds' screening criteria were DL (≥0.18) and OB (≥30%). The TCMSP database was used to screen the targets linked to active ingredients, after which the target information was compared, and the gene name was adjusted using the UniProt database [23]. After matching, 670 targets were discovered, and 201 targets were discovered after removing duplicate values, as shown in Table 4.

**Figure 2:**
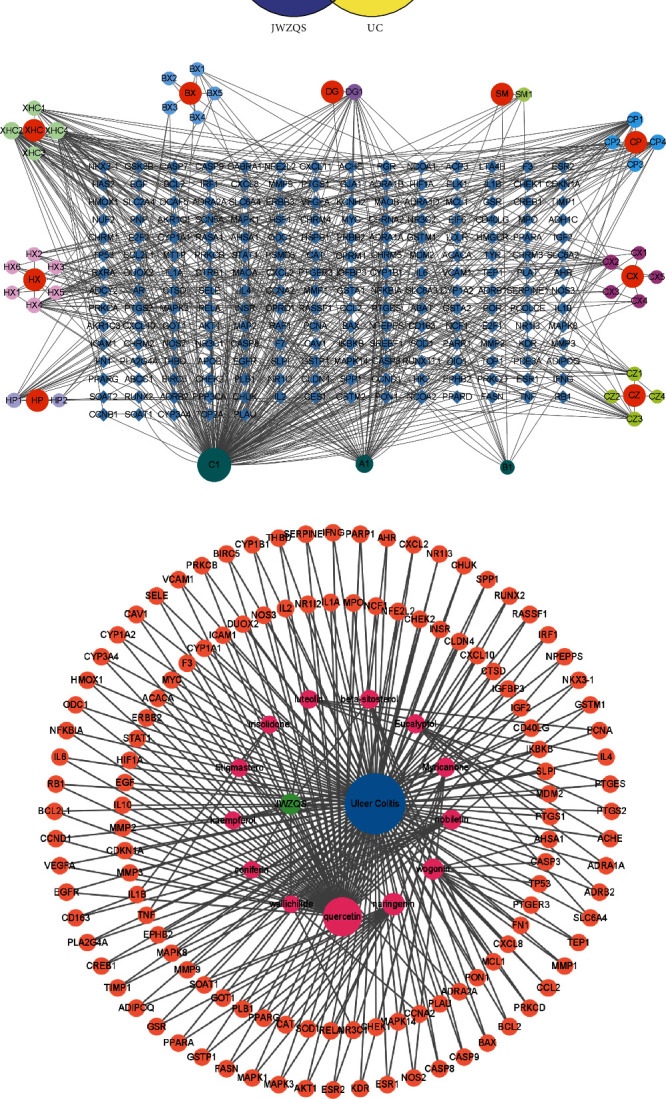
(a) Venn diagram representing the potential targets for JWZQS against UC. (b) “drug-components-targets” network. The orange represents the drug; the surrounding balls represent its components; A1, B1, and C1 represent the common components between drugs; and the diamond represents genes. (c) “Drug-components-targets-disease” network. The blue represents UC, the orange represents the disease target, the green represents JWZQS, and the pink represents the active components.

Using the search term “ulcerative colitis,” the human genome annotation databases GeneCards, OMIM, DisGeNet, and DrugBank were searched for disease-related genes. There were 2127 acquired disease targets. The acquired target genes were compared with the genes associated with the aforementioned drug active ingredients. The String website generated a Venn diagram to screen for common genes, as shown in Figure 2(a).

### 4.2. Construction of “Drug-Components-Targets,” “Drug-Components-Targets-Disease,” and “PPI” Network Maps and Screening of Core Targets

The obtained active ingredient and genetic data will be used to generate “Drug-Components-Targets” and “Drug-Components-Targets-Disease” network maps using Cytoscape software. Figures 2(b) and 2(c) depict the intuitive relationship between drugs, diseases, and targets. Thirteen components, including quercetin, naringenin, wogonin, nobiletin, and irisolidone, were linked to disease targets and may be useful in the treatment of UC.

**Figure 3:**
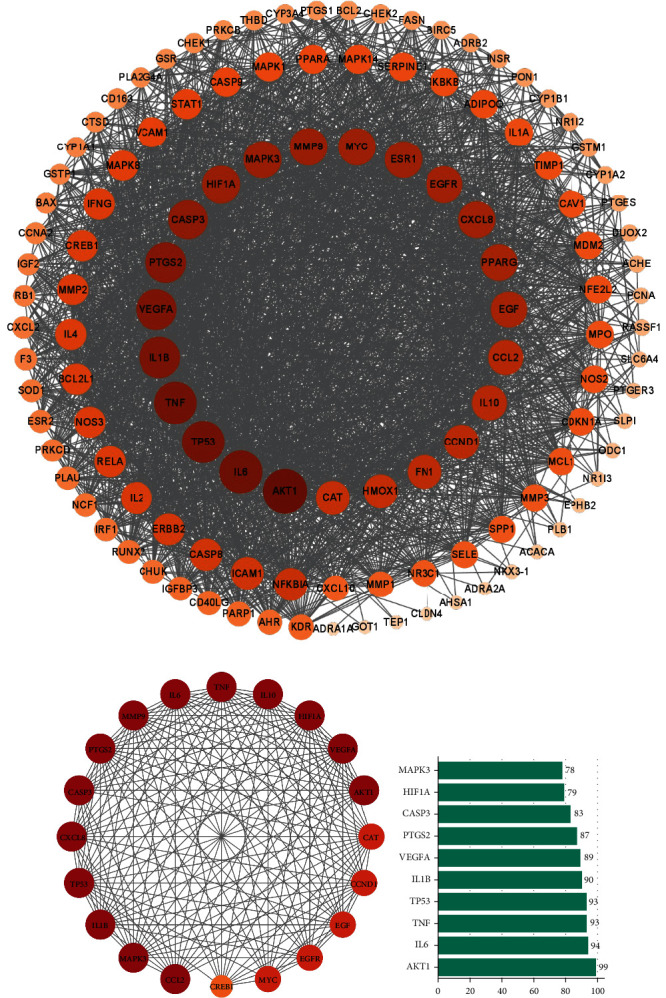
Network diagram showing how UC and JWZQS target proteins interact. (a) PPI network with 123 repeated targets. (b) The network of the 20 core proteins. (c) The rank of correlation “degree” of 10 core targets.

With the common gene data imported, the String database was used to generate a protein-protein interaction network (PPI) interaction map with the species “Homo Sapiens” as the designation. The TSV file was exported with the lowest interaction score (0.7). The Cytoscape software was used to calculate the major protein interactions. We imported the 123 common targets in the intersection into the String database to generate the interaction map. We import the interaction diagram into Cytoscape software to generate the PPI network diagram (Figure 3(a)).

TAccording to the network, the core targets were AKT1, IL6, TP53, TNF, IL-1*β*, VEGFA, TPGS2, CASP3, HIF1A, MAPK3, and other genes, ranked by degree value (Figures 3(b) and 3(c)).

### 4.3. GO and KEGG Analyses

**Figure 4:**
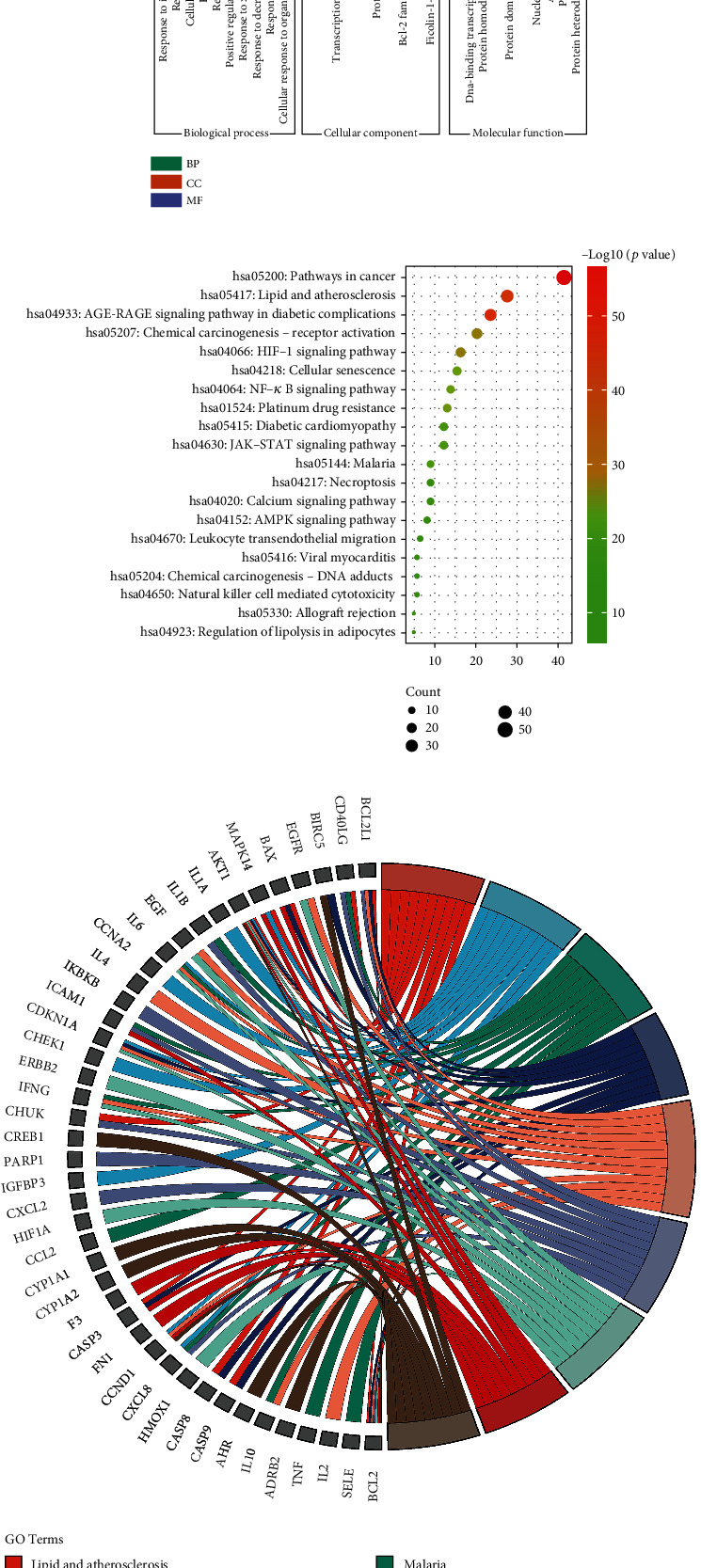
GO analysis of JWZQS in UC treatment. (a) GO analysis, including biological process (BP), molecular functionality (MF), and cellular component (CC). (b) Enrichment bubble diagram of the first 10 pathways. (c) Chord diagrams of the first 10 pathways and corresponding targets.

**Figure 5:**
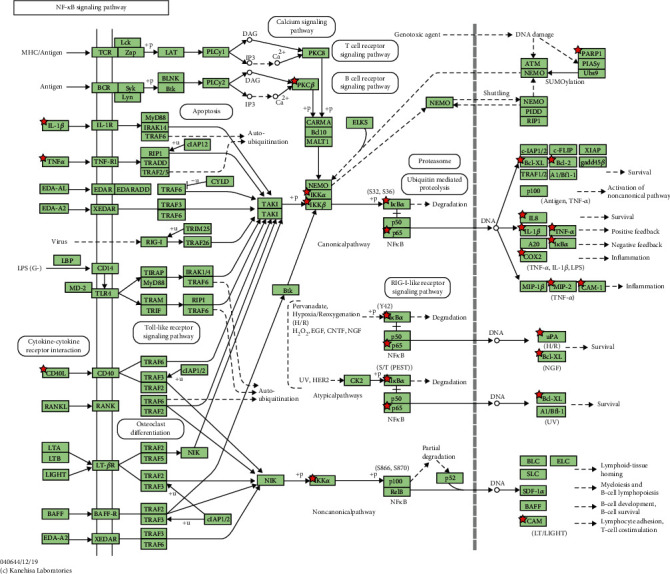
Red nodes represent targets regulated by JWZQS in NF kappa B pathways.

The GO function and KEGG pathway of JWZQS were investigated using Metascape to better understand the compound's treatment mechanism on UC. In terms of BP, the therapeutic effect of JWZQS on UC is mainly due to cellular responses to lipid, hormones, substances, lipid peptides, radiation, positive regulation of cell motility, and other factors. Protein homodimerization activity, cytokine activity, transcription factor binding, protein domain-specific binding, DNA binding, kinase binding, and other activities are all part of the MF project. In terms of CC, JQZQS may influence membrane rafts, transcription regulator complexes, membrane sides, vesicle lumens, and other cell structures. JWZQS may be used to treat UC by interfering with these BP (Figure 4(a)). Cancer, lipid and atherosclerosis, diabetic complications, chemogenic receptor activation, cellular senescence, platinum resistance, and diabetic cardiomyopathy were the most affected pathways in the KEGG analysis (Figure 4(b)). We discovered numerous associations between rich pathways and additional pathological effects, which could be attributed to shared molecular targets in various diseases (Figure 4(c)). JWZQS may regulate the expression of TNF-*α*, IL-6, and IL-1*β* in the NF-*κ*B pathway. It may control the phosphorylation of I*κ*B-*α* and P65 to prevent inflammation (Figure 5).

### 4.4. Molecular Docking

**Figure 6:**
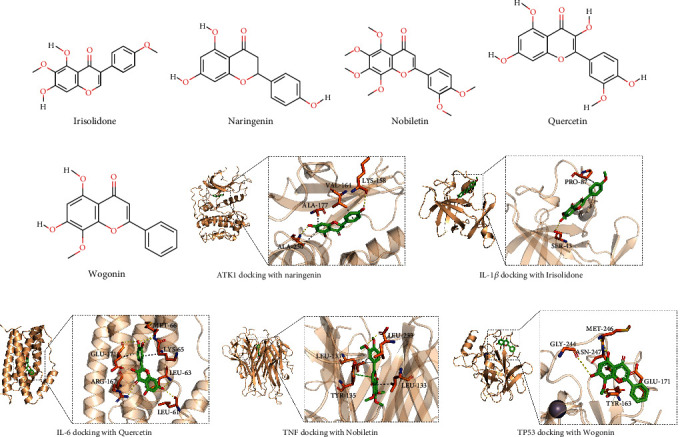
The first five components of JWZQS that play a major role in the treatment of UC. (a) Irisolidone. (b) Naringenin. (c) Nobiletin. (d) Quercetin. (e)Wogonin. (f–j) The results of molecular docking. The picture on the left shows the overall view, while the picture on the right shows the partial view. In the picture, stick is a small molecule in green, while the protein is in orange cartoon. The dotted line in yellow represents hydrogen bonding, and the dotted line in gray represents hydrophobic action. PDB ID: TNF (7JRA), AKT1 (4GV1), IL-6 (7NXZ), IL-1*β* (5R8J), and TP53 (7DHZ).

We discovered in the PPI network diagram that the core targets were AKT1, IL6, TP53, TNF, and IL-1*β*. In the “drug-components-targets-disease” network diagram, quercetin, naringenin, wogonin, nobiletin, and irisolidone are discovered to be the main active components of JWZQS. We used molecular docking to find their corresponding relationship in the drug-components-targets-disease network diagram. As shown in Figure 2(c), Vina 1.1.2 was used to investigate the docking of compounds irisolidone, naringenin, nobiletin, quercetin, and wogonin with IL-1*β*, AKT1, TNF, IL-6, and TP53 proteins, respectively (Figure 6).

TA negative binding affinity indicates the likelihood of binding, and when the affinity value is less than –6 kcal/mol, the binding is frequently thought to be highly likely. Docking scores for AKT1 and naringenin are –8.1 (kcal/mol), IL-1*β* and irisolidone are –6.4 (kcal/mol), IL-6 and quercetin are –7.4 (kcal/mol), TNF and nobiletin are –9.1 (kcal/mol), and TP53 and wogonin are –6.6 (kcal/mol). It is worth noting that AKT1 has a high affinity with naringenin and TNF with nobiletin. Then, we dock all ligands for each docked protein, to observe the interaction between the core targets and other active components (Table 5).

### 4.5. JWZQS Inhibited the Expression of the NF-*κ*B Pathway

**Figure 7:**
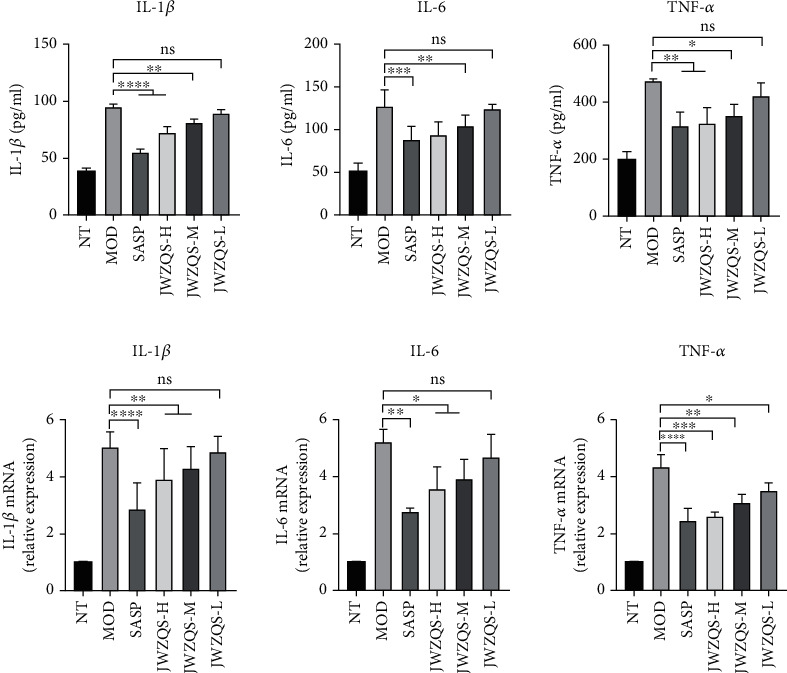
JWZQS inhibits the production of related inflammatory factors. (a) Expression levels of IL-1*β*, IL-6, and TNF-*α* inflammatory factors in mouse colon tissue. (b) mRNA relative expression levels of IL-1*β*, IL-6, and TNF-*α* in mouse colon tissue. Data are means ± SD (*n* = 3). ^∗∗∗∗^*P* < 0.0001, ^∗∗∗^*P* < 0.001, ^∗∗^*P* < 0.01, and ^∗^*P* < 0.05.

**Figure 8:**
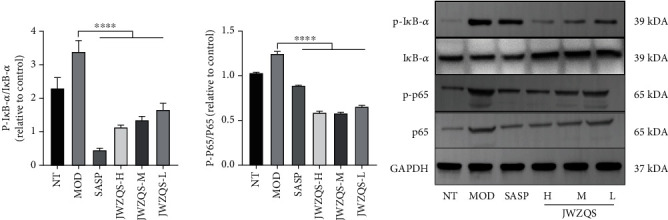
JWZQS effectively controlled the expression of phosphorylated protein in the UC NF-*κ*B signaling pathway. (a) The phosphorylation p65 and I*κ*B-*α* protein in each group was expressed by Western Blot. (b) The protein bands of each group were detected by Western Blot. Data are means ± SD (*n* = 3). ^∗∗∗∗^*P* < 0.0001, ^∗∗∗^*P* < 0.001, ^∗∗^*P* < 0.01, and ^∗^*P* < 0.05.

We examined the levels of inflammatory factors in the colons of mice in each group. We discovered that JWZQS could inhibit the expression of inflammatory factors, such as IL-6, IL-1*β*, and TNF-*α* in the MOD group, effectively controlling inflammation development (Figure 7). Protein bands from the NF-*κ*B pathway were analyzed to investigate the protective mechanism of JWZQS in UC mice (Figure 8(b)). JWZQS effectively inhibited I*κ*B-*α* and P65 phosphorylation in the NF-*κ*B pathway (Figure 8(a)). Therefore, JWZQS can inhibit the NF-*κ*B signaling pathway activation and reduce colon inflammation in mice.

### 4.6. JWZQS Showed a Good Protective Effect on DSS-Induced UC Mouse Model

**Figure 9:**
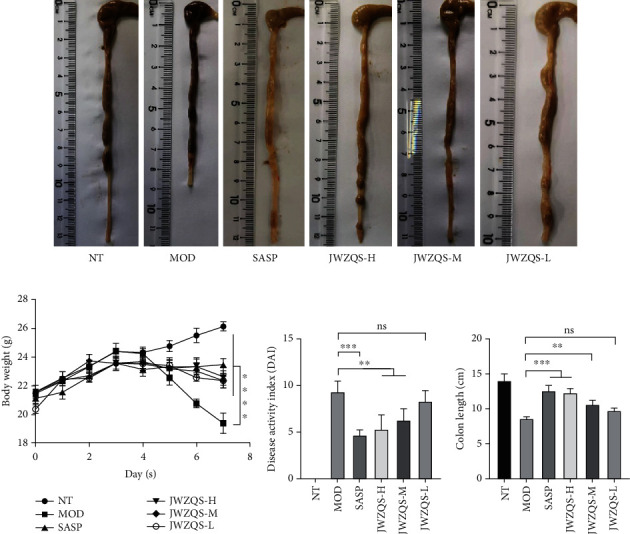
JWZQS effectively alleviated colon injury in UC mice. (a) Example of colon length of mice in each group. (b) Histogram of colon length. (c) Histogram of DAI score. (d) Curve of body weight change of mice. Data are means ± SD (*n* = 3). ^∗∗∗∗^*P* < 0.0001, ^∗∗∗^*P* < 0.001, ^∗∗^*P* < 0.01, and ^∗^*P* < 0.05.

Images of colon samples revealed that fecal urine appeared in the colons of MOD group mice, and the colon length was significantly reduced (Figures 9(a) and 9(b)). Every day, the clinical symptoms of mice were recorded (Figure 9(c)). Every day, except for the blank group, the weight of the mice was recorded (Figure 9(d)). The MOD group's average colon length was shorter than that of the other groups.

**Figure 10:**
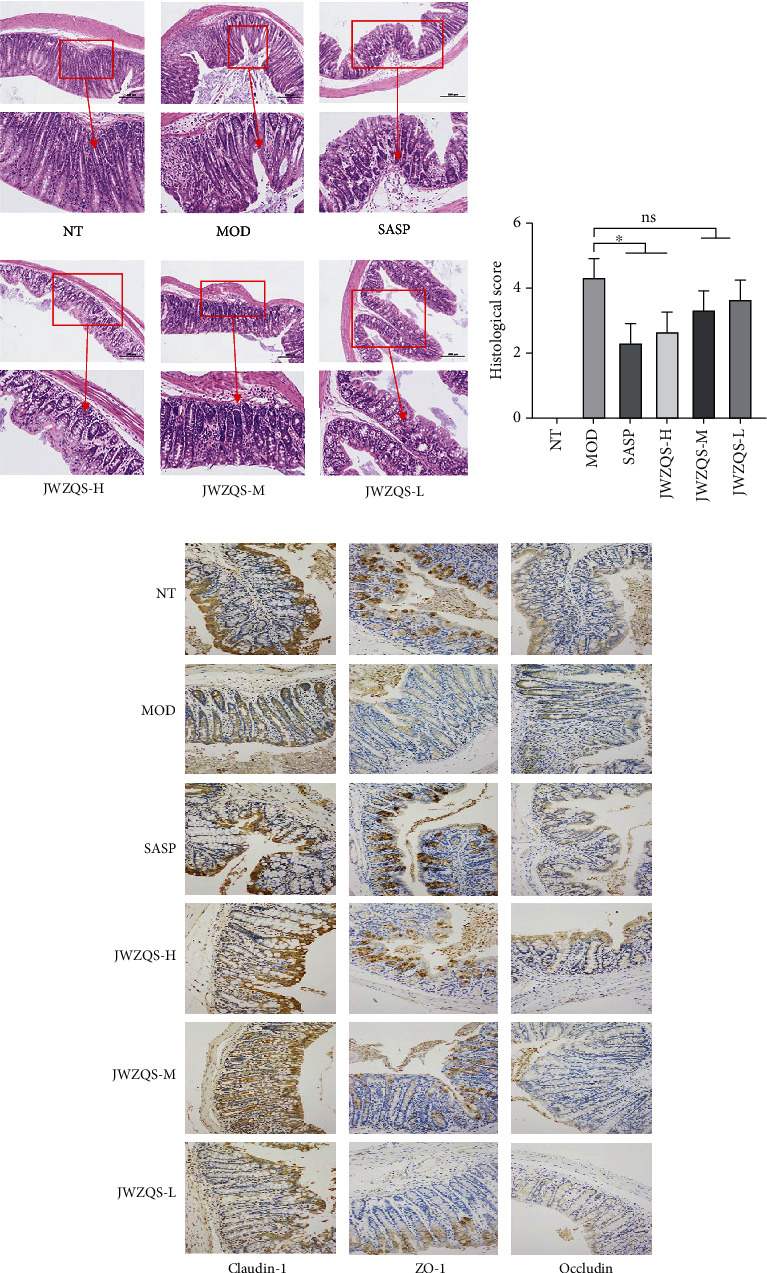
Protective effect of JWZQS on DSS on colonic injury in mice. (a) H&E staining showed colon injury in each group. (b) Histological scores. (c) Expression of Claudin-1, ZO-1, and Occludin in colon tissue of mice in each group. Data are means ± SD (*n* = 3). ^∗∗∗∗^*P* < 0.0001, ^∗∗∗^*P* < 0.001, ^∗∗^*P* < 0.01, and ^∗^*P* < 0.05.

H&E staining was used to stain mouse colon specimens. After staining, the histology was examined under a light microscope, and histological evaluations were carried out. The MOD group mice had fewer colonic goblet cells, lost colonic crypts, and typical edematous infiltration of inflammatory tissue when compared to the other groups (Figure 10(a)). The results revealed that the content of MOD histone decreased significantly, and the SASP group clearly outperformed the MOD group, as did the Chinese medicine dose groups, which also outperformed the MOD group and demonstrated a concentration gradient advantage (Figure 10(b)).

## 5. Discussion

UC is currently classified as a refractory condition by the medical community. Mucous pus, diarrhea, hemafecia, and stomachache are some of the clinical symptoms. Its lesions mostly infiltrate the rectal and colonic mucosa/submucosa. Clinical characteristics include a long disease course, ease of recurrence, a difficult prognosis, and numerous complications [24]. Despite its low prevalence, UC is on the rise, and it is a refractory disease with a lengthy treatment cycle and a high fatality and disability rate [25]. The current oversimplified Western drug therapies are not only costly but also heavily dependent and drug-resistant. Therefore, it is critical to improve treatment methods and encourage the development of new, more effective therapeutic options with fewer side effects.

Along with the growth of network pharmacology, a new method for locating therapeutic agents has recently emerged. The multitarget activity in network pharmacology corresponds to the complex disease pathways and drug action. Understanding TCM through network pharmacology is a growing trend [26]. Network pharmacology is a cutting-edge research method that combines network science, bioinformatics, and systems biology to assess molecular associations between pharmaceuticals and therapeutic entities at the physiological network and system level facets and clarify drug methodological pharmacodynamics. Exploring such intricate TCM constituents is a natural fit for its multichannel and multitarget properties [27]. Therefore, the current study established the mechanism of action of JWZQS in methodologically managing UC.

The analysis of “drug-components-targets” and “drug-components-targets-disease” revealed 13 active ingredients, which are key ingredients in the treatment of UC. The PPI network discovered that AKT1, IL6, TP53, TNF, and IL-1*β* are core targets, and their interactions are critical to the completion of various biological processes. GO and KEGG analyses revealed that JWZQS's anti-UC effect is primarily derived from the cellular response to positive regulation of lipids, hormones, substances, lipid peptides, radiation, and cell movement. Some examples include protein homodimer activity, cytokine activity, transcription factor binding, protein domain-specific binding, DNA binding, kinase binding, and other MF items. JQZQS may influence membrane rafts, transcriptional regulatory complexes, membrane sides, vesicle lumens, and other cellular structures in CC. JWZQS may be used to treat UC by interfering with these biological processes. JWZQS are primarily involved in regulating cancer, lipid and atherosclerosis, diabetic complications, chemogenic receptor activation, cellular senescence, platinum resistance, and diabetic cardiomyopathy. However, TNF-*α*, IL-6, and IL-1*β* may be regulated in the NF-*κ*B pathway. It also influences I*κ*B-*α* and P65 phosphorylation. Animal studies will confirm this later. Irisolidone, naringenin, nobiletin, quercetin, and wogonin are some of the most promising UC treatments. Irisolidone, naringenin, nobiletin, quercetin, and wogonin were found to have affinity for IL-1*β*, AKT1, TNF, IL6, and TP53 proteins. However, the interaction of IL1*β*, IL6, and TP53 with the active constituents was weak in the docking results. There may be the following reasons. IL1*β*, IL6, and TP53 proteins were small and could not form obvious binding pockets. The active ingredient binds only to the surface of the protein. The molecular docking mode is semiflexible docking, which cannot completely simulate the induced fit in body. Molecular docking does not fully simulate the environment in body.

We found that the NF-*κ*B pathway may be involved in the important pathway of JWZQS therapy for UC. The NF-*κ*B pathway regulates the expression of numerous genes that regulate immune responses and inflammation, as well as cellular proliferation, apoptosis, and survival [28, 29]. Dimers are formed when NF-*κ*B/Rel proteins, which are kept inactive by collaborating with I*κ*B repressor proteins and p50 and p52's p105 and p100 precursors combined. The inducer of NF-*κ*B is an IKK complex composed of the NEMO/IKK*γ* regulatory and IKK*α* and IKK*β* catalytic subunits [30–32]. IKK compounds, particularly those with serine residues in protein phosphorylation I*κ*B, predominate in response to various stimuli (e.g., TNF, IL-1*β*, and other proinflammatory cytokines) and bacterial processes, such as fat polysaccharide activation. These compounds target serine residues for significant degradation mediated by the ubiquitin-proteasome, resulting in the release of NF-*κ*B, nuclear accumulation, and transcriptional activation of target genes [33, 34]. Because a variety of cell types are involved in the pathogenesis of the disease that resembles IBD, the intracellular functionalities of NF-*κ*B differ by cell type. Increased proinflammatory mediator levels are most likely caused by increased NF-*κ*B immunocyte activity, which worsens the inflammation. Therefore, we looked into how JWZQS affected the phosphorylation of P65 and I*κ*B-*α* proteins in the NF-*κ*B signaling pathway in animals. JWZQS inhibits the activation of the NF-*κ*B signaling pathway, which inhibits the release of inflammatory cytokines IL-1*β*, IL-6, and TNF-*α* in a dose-dependent manner.

In this study, it was discovered that JWZQS not only had a protective effect on pathological injury and colon shortening throughout the DSS induced UC mouse model but also significantly improved body weight maintenance in mice. JWZQS was dose-dependent in UC mice, according to the findings. The breakdown of the intestinal immune barrier, which is formed by tight connections between cells, is a key factor in the progression of UC. Occludin, Claudin-1, and ZO-1 are intestinal immune barrier-tight junction proteins that are primarily responsible for maintaining the integrity of the intestinal epithelial barrier. Increased permeability of these junction proteins is associated with an increased risk of UC [35]. The study of these three tight junction proteins revealed that JWZQS had a protective effect on the intestinal immune barrier, indicating that the expression levels of Occludin, Claudin-1, and ZO-1 were lower in the DSS-induced UC mouse model than in the other groups. JWZQS could significantly correct the abnormal expression of three tight junction proteins in UC model mice, effectively reduce cell tissue permeability, repair intestinal mucosa, and alleviate UC symptoms.

These results suggest that JWZQS treat UC by inhibiting the expression of TNF-*α*, IL-6, and IL-1*β* in the NF-*κ*B pathway. JWZQS protects DSS-induced UC mouse models by upregulating Occludin, Claudin-1, and ZO-1 in the colon of mice. In addition, many additional signaling pathways may be activated and their mechanisms remain to be investigated.

## 6. Conclusion

In this study, we used network pharmacology and related software system to study the potential mechanism of JWZQS against UC. The affinity between the core target and the active component was verified by molecular docking. Animal experiments revealed that JWZQS could inhibit the NF-*κ*B pathway, reduce the expression of related inflammatory factors, and increase the expression of tight link protein. However, additional research is required to validate the reliability of the research findings. Finally, JWZQS can be used in the clinic. However, the mechanism of JWZQS in treating UC requires more research.

## Figures and Tables

**Table 1:** Disease activity index (DAI).

Score	Bodyweight decrease rate	Fecal property	Hematochezia status
0	0%	Normal	Normal
1	1–5%	Semi loose (+)	Feces with occult blood (+)
2	6–10%	Semi loose (++)	Feces with occult blood (++)
3	11–15%	Loose (+)	Bloody feces (+)
4	>15%	Loose (++)	Bloody feces (++)

**Table 2:** Primers utilized in qRT-PCR.

NM_008084.2	M-GAPDH-S	CCTCGTCCCGTAGACAAAATG
	M-GAPDH-A	TGAGGTCAATGAAGGGGTCGT
NM_001278601.1	M-TNF*α*(4)-S	CCCTCACACTCACAAACCACC
	M-TNF*α*(4)-A	CTTTGAGATCCATGCCGTTG
NM_001314054.1	M-IL6(1)-S	AGTTGTGCAATGGCAATTCTGA
	M-IL6(1)-A	CTCTGAAGGACTCTGGCTTTGTC
NM_008361.4	M-IL1B-S	GCATCCAGCTTCAAATCTCGC
	M-IL1B-A	TGTTCATCTCGGAGCCTGTAGTG

**Table 3:** Histological scoring criteria.

Score	Mucosal architecture	Cellular infiltration	Goblet cell depletion
0	Absent	None	Absent
1	Mild	Infiltrate around the crypt basis	Present
2	Medium	Infiltrate reaching the muscularis mucosae	
3	Severe	Infiltrate reaching the submucosa	

**Table 4:** Active ingredients of herbs as identified by the UniProt database.

Latin name	MOL ID	Active ingredient	OB ≥ 30%	DL ≥ 0.18	ID
Magnolia officinalis Rehd Et Wils.	MOL005970	Eucalyptol	60.62	0.32	HP1
Magnolia officinalis Rehd et Wils.	MOL005980	Neohesperidin	57.44	0.27	HP2
Atractylodes lancea (Thunb.) DC.	MOL000173	Wogonin	30.68	0.23	CZ1
Atractylodes lancea (Thunb.) DC.	MOL000184	NSC63551	39.25	0.76	CZ2
Atractylodes lancea (Thunb.) DC.	MOL000188	3*β*-Acetoxyatractylone	40.57	0.22	CZ3
Atractylodes lancea (Thunb.) DC.	MOL000085	Beta-daucosterol_qt	36.91	0.75	CZ4
Angelicae Sinensis Radix	MOL000358	Beta-sitosterol	36.91	0.75	DG1
Angelicae Sinensis Radix	MOL000449	Stigmasterol	43.83	0.76	A1
Arum ternatum Thunb.	MOL000519	Coniferin	31.11	0.32	BX1
Arum ternatum Thunb.	MOL006936	10,13-Eicosadienoic	39.99	0.2	BX2
Arum ternatum Thunb.	MOL006957	Piperazine-2,5-quinone	46.89	0.27	BX3
Arum ternatum Thunb.	MOL003578	Cycloartenol	38.69	0.78	BX4
Arum ternatum Thunb.	MOL006967	Beta-D-Ribofuranoside, xanthine-9	44.72	0.21	BX5
Chuanxiong	MOL001494	Mandenol	42	0.19	CX1
Chuanxiong	MOL002135	Myricanone	40.6	0.51	CX2
Chuanxiong	MOL002140	Perlolyrine	65.95	0.27	CX3
Chuanxiong	MOL002157	Wallichilide	42.31	0.71	CX4
Chuanxiong	MOL000359	Sitosterol	36.91	0.75	B1
Chuanxiong	MOL000433	FA	68.96	0.71	CX5
Cimicifugae Rhizoma	MOL000449	Stigmasterol	43.83	0.76	A1
Cimicifugae Rhizoma	MOL000483	Acrylamide	118.35	0.26	SM1
Citrus reticulata	MOL000359	Sitosterol	36.91	0.75	B1
Citrus reticulata	MOL004328	Naringenin	59.29	0.21	CP1
Citrus reticulata	MOL005100	Chroman-4-one	47.74	0.27	CP2
Citrus reticulata	MOL005815	Citromitin	86.9	0.51	CP3
Citrus reticulata	MOL005828	Nobiletin	61.67	0.52	CP4
Pogostemon cablin (Blanco) Benth.	MOL002879	Diop	43.59	0.39	HX1
Pogostemon cablin (Blanco) Benth.	MOL005573	Genkwanin	37.13	0.24	HX2
Pogostemon cablin (Blanco) Benth.	MOL005911	5-Hydroxy-7,4′-dimethoxyflavanon	51.54	0.27	HX3
Pogostemon cablin (Blanco) Benth.	MOL005916	Irisolidone	37.78	0.3	HX4
Pogostemon cablin (Blanco) Benth.	MOL005918	Phenanthrone	38.7	0.33	HX5
Pogostemon cablin (Blanco) Benth.	MOL005921	Quercetin 7-O-*β*-D-glucoside	49.57	0.27	HX6
Pogostemon cablin (Blanco) Benth.	MOL000098	Quercetin	46.43	0.28	C1
Agrimonia eupatoria	MOL001002	Ellagic acid	43.06	0.43	XHC1
Agrimonia eupatoria	MOL000422	Kaempferol	41.88	0.24	XHC2
Agrimonia eupatoria	MOL000492	(+)-Catechin	54.83	0.24	XHC3
Agrimonia eupatoria	MOL000006	Luteolin	36.16	0.25	XHC4
Agrimonia eupatoria	MOL000098	Quercetin	46.43	0.28	C1

**Table 5:** Docking_score (kcal/mol).

Target_name	Ligand_name	Docking_score (kcal/mol)
AKT1	Irisolidone	-7.8
AKT1	Nobiletin	-7.2
AKT1	Quercetin	-8.1
AKT1	Wogonin	-7.6
IL-1*β*	Naringenin	-7.1
IL-1*β*	Nobiletin	-6.3
IL-1*β*	Quercetin	-6.8
IL-1*β*	Wogonin	-6.8
IL-6	Irisolidone	-6.1
IL-6	Naringenin	-7.5
IL-6	Nobiletin	-6
IL-6	Wogonin	-6.4
TNF	Irisolidone	-8.9
TNF	Naringenin	-8.7
TNF	Quercetin	-8.7
TNF	Wogonin	-9.1
TP53	Irisolidone	-6.7
TP53	Naringenin	-6.7
TP53	Nobiletin	-6.1
TP53	Quercetin	-7.2

## Data Availability

The data used to support the findings of this study are available from the corresponding author upon request.
